# Strong positive effects of termites on savanna bird abundance and diversity are amplified by large herbivore exclusion

**DOI:** 10.1002/ece3.3513

**Published:** 2017-10-23

**Authors:** Stein R. Moe, Katrine Eldegard, Ole Tobias Rannestad, Paul Okullo, Ommund Lindtjørn, Ole Gunnar Støen, Svein Dale

**Affiliations:** ^1^ Faculty of Environmental Sciences and Natural Resource Management Norwegian University of Life Sciences P.O. Box 5003, NO‐1432 Ås Norway; ^2^ Nabuin Zonal Agricultural Research and Development Institute National Agricultural Research Organisation Moroto Uganda

**Keywords:** bird diversity, ecological facilitation, *Macrotermes*, savanna dynamics, ungulates

## Abstract

Vast areas of the African savanna landscapes are characterized by tree‐covered *Macrotermes* termite mounds embedded within a relatively open savanna matrix. In concert with termites, large herbivores are important determinants of savanna woody vegetation cover. The relative cover of woody species has considerable effects on savanna function. Despite the potentially important ecological relationships between termite mounds, woody plants, large herbivores, and birds, these associations have previously received surprisingly little attention. We experimentally studied the effects of termites and large herbivores on the avian community in Lake Mburo National Park, Uganda, where woody vegetation is essentially limited to termite mounds. Our experiment comprised of four treatments in nine replicates; unfenced termite mounds, fenced mounds (excluding large mammals), unfenced adjacent savanna, and fenced savanna. We recorded species identity, abundance, and behavior of all birds observed on these plots over a two‐month period, from late dry until wet season. Birds used termite mounds almost exclusively, with only 3.5% of observations occurring in the treeless intermound savanna matrix. Mean abundance and species richness of birds doubled on fenced (large herbivores excluded) compared to unfenced mounds. Feeding behavior increased when large mammals were excluded from mounds, both in absolute number of observed individuals, and relative to other behaviors. This study documents the fundamental positive impact of *Macrotermes* termites on bird abundance and diversity in an African savanna. Birds play crucial functional roles in savanna ecosystems, for example, by dispersing fruits or regulating herbivorous insect populations. Thus, the role of birds in savanna dynamics depends on the distribution and abundance of termite mounds.

## 1 INTRODUCTION

Savanna ecosystems are characterized by a continuous layer of herbaceous plants with large spatial variations in a discontinuous woody cover. The extent of woody vegetation cover has considerable positive impact on the avifauna (Gottschalk, Ekschmitt, & Bairlein, 2007; Herremans, 1995). Rainfall plays a role in limiting the upper level of tree density, with herbivory, fire, and soil nutrients contributing to tree covers below maximum densities (Sankaran et al., 2005).

Mounds constructed by *Macrotermes* termites (family Termitidae) are an important source of savanna heterogeneity. Across African savannas, large mounds, sometimes covered by dense woody vegetation, can be readily seen in satellite imagery and aerial photographs (e.g., Bonachela et al., 2015, Google Earth 2017). The large nutrient‐rich mounds (Lee & Wood, 1971) are spatially overdispersed (Pringle, Doak, Brody, Jocque, & Palmer, 2010), often in a grassland‐dominated savanna matrix, and in many parts of Africa, large *Macrotermes* mounds are covered with dense woody vegetation (Levick, Asner, Kennedy‐Bowdoin, & Knapp, 2010). *Macrotermes* termites are providers of key ecosystem services (see Jouquet, Traoré, Choosai, Hartmann, & Bignell, 2011 for a review). Their role and importance as ecosystem engineers in tropical ecosystems are functionally similar to earthworms in temperate areas (Petersen & Luxton, 1982). Termites are important for soil turnover (De Bruyn & Conacher, 1990) and in symbiosis with *Termitomyces* sp. fungi, *Macrotermes* termites transform organic material into plant‐available inorganic forms, creating favorable sites for tree and forb establishment (Van der Plas, Howison, Reinders, Fokkema, & Olff, 2013). Trees growing on the mounds are also less affected by seasonal floods (Jouquet et al., 2011), and by fires, because mounds are slightly elevated compared to the fire‐prone grass‐dominated matrix, their footslopes are often covered by bare soil, mounds have humid soil and foraging by herbivores reduce fuel load (Bloesch, 2008; Joseph, Seymour, Cumming, Mahlangu, & Cumming, 2013). The mound vegetation has distinct tree species composition and functional characteristics, relative to the trees growing in the surrounding savanna matrix (Van der Plas et al., 2013). Termite mounds are nutrient hot spots changing the spatial distribution of resources on a landscape scale (Jouquet et al., 2011). Mounds are hot spots, not only for vegetation, but also for a wide range of other taxa (e.g., Fleming & Loveridge, 2003; Holdo & McDowell, 2004; Loveridge & Moe, 2004; Okullo, Greve, & Moe, 2013).

Large ungulate herbivores are also a key functional group in African savannas (e.g., Asner et al., 2009). Large mammalian browsers directly affect woody cover and tree species composition through selective feeding on trees, particularly seedlings (Moe, Rutina, Hytteborn, & du Toit, 2009; Støen, Okullo, Eid, & Moe, 2013). Conversely, large mammalian grazers can facilitate tree regeneration by reducing competition with grasses, decreasing fire frequency, and altering the vegetation, indirectly reducing populations of small mammalian seed and seedling predators (Goheen, Palmer, Keesing, Riginos, & Young, 2010).

Birds can be important contributors to ecosystem resilience and function (Sekercioglu, 2006) and have been termed mobile links because they connect habitats in space and time (Lundberg & Moberg, 2003). Birds can influence plant survival and reproduction, both as seed predators (Kelt, Meserve, & Gutiérrez, 2004) and as regulators of herbivorous insects (Van Bael, Brawn, & Robinson, 2003). Many frugivorous birds are also important seed dispersers, dispersing seeds among focal feeding sites (Nogales, Delgado, & Medina, 1998) such as termite mounds (Yamashina, 2014).

Only a few studies have looked at interactions between termites and birds. Woody plants in termite mounds provide nesting, feeding, and perching sites for birds (Brightsmith, 2000; Kesler & Haig, 2005; Sanchez‐Martinez & Renton, 2009; Vasconcelos, Hoffmann, Araújo, & Vasconcelos, 2015). Both the diversity and abundance of cavity‐nesting birds are significantly correlated with number of termite mounds in miombo woodlands (Joseph et al., 2011), where savanna matrix tree densities are relatively high.

We also have limited knowledge of how large savanna herbivores affect bird abundance and diversity. High densities of large wild mammals, which substantially affect vegetation structure and composition (Sankaran, Augustine, & Ratnam, 2013), characterize savannas in Africa. In Kenya, Ogada, Gadd, Ostfeld, Young, and Keesing (2008) documented a 30% increase in bird diversity in plots from which large mammals were excluded, and in southern Africa, intensive browsing by elephant (*Loxodonta africana*) reduced bird species abundance and diversity (Cumming et al., 1997; Herremans, 1995). We know of no studies that have attempted to disentangle the combined effects of large ungulates and termites on savanna bird abundance, richness, and diversity. Such knowledge is important for fully understanding spatial variability in savanna structure and temporal variation in function.

Large vegetated termite mounds built by *Macrotermes* species are a conspicuous feature of the savanna landscape in Lake Mburo National Park (LMNP), Uganda (Okullo & Moe, 2012a). The mounds occupy only 5% of the landscape, but they explain 89% of the distinct patches with dense woody vegetation (Bloesch, 2008; Moe, Mobæk, & Narmo, 2009). Although the density of the mounds may be low in many savanna areas, termite mounds can influence browsing patterns over as much as 20% of the savanna landscape (Levick et al., 2010). In an East Africa savanna, it has been shown that pattern‐generating organisms, such as termites, are central in biomass accumulation and govern ecosystem functions such as N_2_ fixation (Fox‐Dobbs, Doak, Brody, & Palmer, 2010; Pringle et al., 2010). Previous studies have shown that *Macrotermes* mounds in LMNP are associated with distinct species assemblages of herbaceous (Okullo & Moe, 2012b) and woody vegetation (Bloesch, 2008; Moe, Mobæk et al., 2009); in particular, they harbor virtually all the woody plants and far more forbs in this system.

We studied the influence of interactions between termites and large herbivores on the bird community in Lake Mburo National Park. Because the termite mounds are resource‐rich areas with diverse and dense forb and woody‐dominated vegetation, compared with the grass‐dominated savanna matrix (Okullo & Moe, 2012b), we predicted that termite mounds would have a greater abundance, species richness, and diversity of birds. We hypothesized that large ungulate exclusion would increase the overall abundance and diversity of birds (see Ogada et al., 2008). Finally, we predicted that frugivorous and nectarivorous birds in particular would benefit from herbivore exclusion because browsers can clip plant shoots and reduce flower and fruit production (Wilkerson, Roche, & Young, 2013). Herbivory may also induce stress responses in plants, altering resource allocation from flower and fruit production to plant secondary metabolites (Boege & Marquis, 2005). Consequently, because of high quantity and quality of resources on fenced mounds, frugivores and nectarivores should be more common and spend more time feeding than on unfenced mounds.

## 2 MATERIALS AND METHODS

### 2.1 Study area

Our study plots (00°32′–00°37′S, 30°55′–31°01′E) were located within Lake Mburo National Park, Uganda, at elevations between 1,200 and 1,300 m above sea level. The average annual rainfall in the area is around 800 mm, with two wet seasons; one from March through May and one from September through November. June and July are the driest months. Average monthly temperatures in the nearby town of Mbarara (1,400 m a.s.l.) range from 19.8°C to 20.9°C ( www.en.climate-data.org). The vegetation in LMNP consists of relatively dry savanna, termitaria‐associated thickets, mixed woodlands, and swamps (Bloesch, 2008).

**Figure 1:**
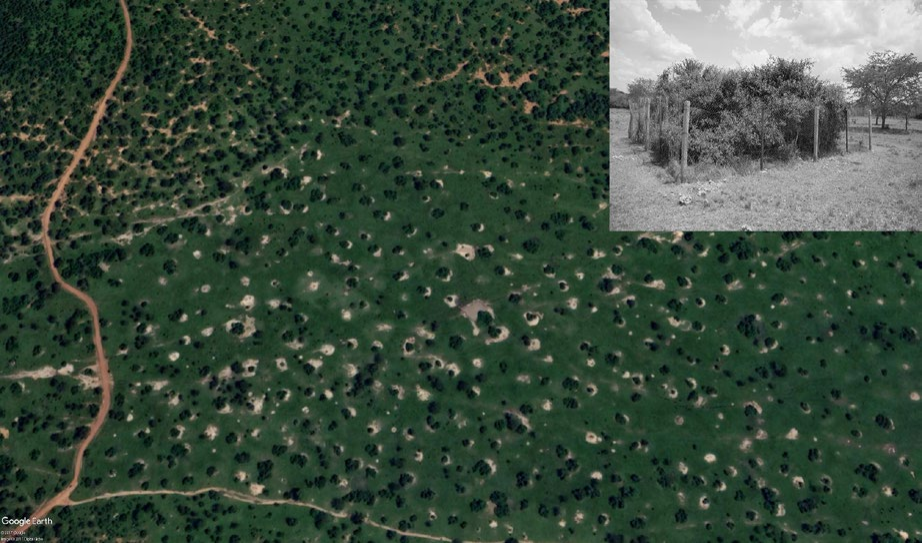
Google Earth (2017) image of Lake Mburo National Park. The distinct termite mounds covered with woody vegetation can be clearly distinguished from the surrounding savanna matrix. The small photograph shows one of the fenced termite mounds

Large termite mounds (5‐10 m diameter) in LMNP are scattered in a matrix of treeless, grass‐dominated savanna (Figure 1). Mounds are constructed by *Macrotermes subhyalinus* (D. E. Bignell pers. com.). This species is morphologically similar to *M. herus*, but it is possible that *M. subhyalinus* and *M. herus* will be considered one species in a future evaluation of the genus (D. E. Bignell, pers. com.).

Tree densities are four times as high on termite mounds as the savanna matrix (Table 1). *Rhus natalensis*,* Scutia myrtina,* and *Grewia similis* are common on mounds, while different acacia species (i.e., *A. gerrardii, A. sieberiana,* and *A. hockii*) are the most common tree species on the savanna matrix. Forbs, such as *Psilotrichum axilliflorum* and *Commelina africana,* are generally associated with termite mounds (Okullo & Moe, 2012b). Grasses, particularly *Sporobolus pyramidalis* and *Brachiaria decumbens,* are the dominant herbaceous plants on both savanna and termite mounds (Okullo & Moe, 2012b). The termite mound vegetation acts as a feeding hot spot for large herbivores (Mobæk, Narmo, & Moe, 2005) and supports an abundance of small mammals (Okullo et al., 2013).

**Table 1:** Vegetation (trees > 0.5 m) characteristics (mean ± *SD*) of unfenced savanna (US), fenced savanna (FS), unfenced mound (UM), and fenced mound (FM)

	Treatment			
	US	FS	UM	FM
No. of tree species per plot	2.44 ± 1.51	3.22 ± 2.64	9.78 ± 2.64	9.44 ± 5.10
Trees m^−2^	0.06 ± 0.06	0.08 ± 0.07	0.24 ± 0.13	0.27 ± 0.21
Mean tree height (m)	1.15 ± 1.24	1.17 ± 1.22	2.27 ± 1.52	2.45 ± 1.78

The most common wild large herbivores in the area are impala (*Aepyceros melampus*), zebra (*Equus burchelli*), warthog (*Phacochoerus africanus*), waterbuck (*Kobus ellipsiprymnus defassa*), eland (*Tragelaphus oryx*), bushbuck (*Tragelaphus scriptus*), and topi (*Damaliscus lunatus jimela*). The biomass of these species has previously been estimated at 87 kg/ha (Rannestad, Danielsen, Moe, & Stokke, 2006).

### 2.2 Experimental setup

We used an ongoing experiment in LMNP (Okullo & Moe, 2012b) to study the influence of interactions between termites and large herbivores on the bird community. The experiment comprises four treatments; unfenced savanna, fenced savanna, unfenced termite mound, and fenced mound. The fencing excludes large herbivorous mammals.

Nine sites were identified by randomly selecting a compass bearing and a distance from park roads in three locations (i.e., three sites in each of three locations of the park), 6–15 km apart, in June–July 2005. At each site, we selected two termite mounds and two adjacent similar‐sized savanna plots. One each of the two mounds and the two savanna plots were randomly assigned to be fenced to exclude large mammals. Thus, each of the nine sites comprised of four treatment plots: unfenced mound, fenced mound, unfenced savanna, and fenced savanna. Fences were 2 m high, made of 5‐cm galvanized iron mesh to exclude mammalian herbivores larger than lagomorphs. The treatment plots varied from 90 m^2^ to 260 m^2^, depending on the size of the mounds. Within a site, all four treatment plots were the same size. Distance between plots was 20–80 m. Only termite mounds that were active at the onset of the experiment were included. No active or inactive *Macrotermes* mounds were present in the savanna plots. All fenced plots were easily accessible for birds, with the possible exception of large, primarily ground‐dwelling species in the families *Numididae* (guineafowls), *Phasianidae* (spurfowl and francolins), and *Otididae* (bustards). No birds in these families were observed during the study, either in the unfenced or in the fenced plots.

### 2.3 Bird surveys

We recorded species identity, abundance, and behavior of all birds observed on these plots over a two‐month period, from 23 February 2007 (the late dry season) until 18 April 2007 (the wet season). Each data collection period (observation session) for a given plot lasted 30 min. All termite mound plots were observed ten times each, a total of 2,700 min observation for each mound treatment (i.e., 9 mounds × 10 observation sessions × 30 min). Because savanna plots yielded few bird records, we stopped observing these plots after six replicates. This still produced 1,620 min of observation for both treatments (i.e., 9 savanna plots × 6 observation sessions × 30 min). Data collection was performed between 07:00 and 18:30, with a break from 12:00 to 16:00, because most bird species are less active at that time of the day. At each plot, these observation sessions covered different times of the day. Recordings were made by a skilled observer standing in a hidden position 30–40 m away from a given plot, using handheld binoculars and a spotting scope. At the start of every observation session, birds already present were recorded. All additional birds landing inside the plot during the observation period were then monitored. Birds were identified to species level. When possible, bird behavior (i.e., locomoting, perching, preening, feeding, fighting, singing, feeding offspring, nesting, or visiting a nest) was recorded. Birds flying over a plot were not included in the dataset unless they clearly showed signs of foraging in the air space immediately above (<2 m) the plot. Bird nomenclature follows IOC World Bird List, version 6.2 ( http://www.worldbirdnames.org/ioc-lists/family-index/).

### 2.4 Feeding guilds

We subdivided bird species into feeding guilds; *frugivores, granivores*,* nectarivores,* and *insectivores*. The species were assigned to these guilds based mainly on published data (Fry & Keith, 2004; Fry, Keith, & Urban, 1988, 2002; Keith, Urban, & Fry, 1992; Urban, Fry, & Keith, 1986, 2002), but also on our personal experiences in the field.

### 2.5 Data analysis

#### 2.5.1 Bird abundance, species richness, and diversity

Because birds were virtually absent from savanna plots, we restricted further analyses to the termite mounds. To assess whether abundance (number of individual birds), species richness (number of bird species), and bird diversity (Shannon index) differed between fenced and unfenced plots on termite mounds, we used bird observations from each 30‐min observation session as input data. We fitted generalized linear mixed models (GLMM) with abundance, richness, and diversity as response variables, respectively. For each response variable, the full model included the explanatory variables treatment (fencing), tree richness and date (period), and the treatment x period interaction. Observation sessions were clustered in time, and therefore, date was converted into a categorical variable “period” with five levels; [1]: 23 February–1 March, [2]: 6–9 March, [3]: 12–16 March, [4]: 20–24 March, and [5]: 17–18 April. Tree richness was strongly correlated with tree density ([fenced]: *r* = 0.77; [unfenced]: *r* = 0.73). To avoid collinearity, only tree richness was included as explanatory variable. In addition, the full model for each response variable included the interaction term treatment × period. To account for among and within‐sites differences in environmental conditions, treatment plots nested within site and location were included as random effects. We also included the following random effects: observer identity, time of day, and weather. For bird abundance and species richness, we fitted a GLMM with log‐link function, assuming a Poisson distribution of errors. These models were checked for overdispersion by inspecting the generalized Pearson statistic (Crawley, 2013). For richness, the final model, after backward elimination of nonsignificant terms (*p *>* *.05), was not overdispersed (i.e., the gPs value of 0.98 was close to 1). For abundance, the initial model was overdispersed (gPs = 2.5), so we refitted the model with a negative binomial distribution of errors and a log‐link function. For bird diversity, we fitted a GLMM with an identity link function, assuming a normal distribution of errors. To check whether the statistical models provided adequate fit to the observed data, all models were graphically validated with techniques recommended by Zuur, Hilbe, and Ieno (2013).

#### 2.5.2 Bird behavior

For the analyses of whether fencing influenced bird behavior, the input data were the number of bird observations in each behavior category (perching, feeding, territorial, locomotion, preening, parental)—on fenced and unfenced mounds, respectively—summed over all the locations, sites, plots, and observation sessions. In cases of repeated observations of the same individual within the same observation session (i.e., individuals changing behavior), only the first entry (behavior) of the individual was included in the analyses. Parental behavior included feeding offspring, nesting, and visiting nest. Territorial behavior included fighting and singing. To test whether the distribution of behaviors was the same for fenced and unfenced mounds, we carried out a Fisher's exact test on a 2 × 6 (treatment × behavioral category) contingency table.

#### 2.5.3 Community composition and feeding guilds

To test whether the distribution of individuals in different feeding guilds was the same for fenced and unfenced mounds, we carried out a Fisher's exact test on a 2 × 4 (treatment × feeding guild) contingency table. The input data were the number of individuals observed in each feeding guild category and treatment summed over all locations, sites, plots, and observation sessions.

To test whether treatment increased the abundance of birds within each of the feeding guilds, we fitted GLMMs using the same approach and explanatory variables as described above for the analyses of bird abundance and richness.

Finally, to assess whether treatment, tree richness, and tree density were associated with bird community composition, we used nonmetric multidimensional scaling (NMDS) based on a Bray–Curtis dissimilarity matrix calculated from the total (untransformed) abundances of each species in each plot (i.e., summed over ten 30‐min observation sessions). Transforming the bird community data using NMDS allowed community composition to be represented in a few informative dimensions. The coordinates of the sampling sites along a chosen number of axes formed the new variables. We used the metaMDS function in the vegan package in R (Oksanen et al., 2015), with 100 random starting points. The squared correlation coefficient (*R* ^2^) was used to define the relationship between the environmental gradients and NMDS axes scores. The importance of each environmental vector (tree richness, tree density) was assessed from the *R* ^2^ between the scaled environmental variable and the NMDS axes, and the significance of treatment was assessed using *R* ^2^ as a goodness‐of‐fit statistic. The statistical significance (*p*‐values) of both vector and factor variables was based on random permutations of the data. We performed NMDS ordinations of progressively higher dimensions (*k *=* *2 to *k *=* *5), which resulted in stress values of, respectively, 0.22, 0.15, 0.10, and 0.077. In order to reduce complexity, we opted to represent our data in three dimensions (linear fit: *R* ^2^ = 0.78). We used R 3.2.5 software to run all the analyses (R Core Team, 2016).

## 3 RESULTS

### 3.1 Termite mounds versus savanna

**Figure 2:**
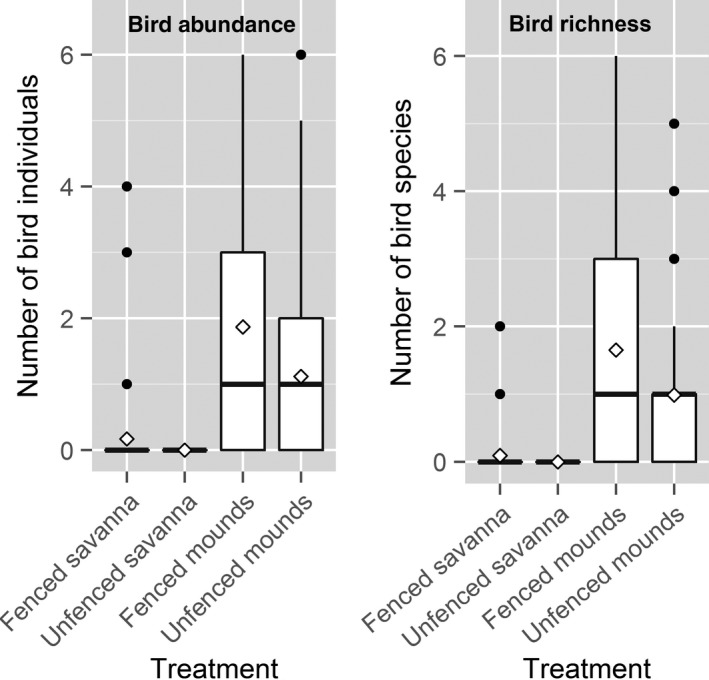
Boxplots for observed values of bird richness and abundance, as recorded during 30‐min observation sessions on fenced and unfenced plots on termite mounds and in savanna. Horizontal black lines show median, and diamonds show mean numbers per 30‐min observations sessions per plot

Birds were almost exclusively associated with the termite mounds (Table 2, Figure 2, Table S1). We recorded 274 individuals of 40 species and 218 individuals of 39 species on fenced and unfenced mounds, respectively (*n* = 90 observation sessions). On the savanna plots (*n* = 54 observation sessions), we recorded 18 individuals of four species in the fenced plots, but none in the unfenced plots (Table 2).

**Table 2:** The abundance and total number of bird species observed on unfenced savanna (US), fenced savanna (FS), unfenced mound (UM), and fenced mound (FM), grouped by feeding guild (see Table S1 for a complete list of species)

Guild	Treatment			
	US	FS	UM	FM
Frugivores				
Abundance	0	0	53	71
No. species	0	0	5	6
Granivores				
Abundance	0	0	19	24
No. of species	0	0	6	7
Insectivores				
Abundance	0	18	124	132
No. of species	0	4	24	23
Nectarivores				
Abundance	0	0	21	42
No. of species	0	0	3	3
Other				
Abundance	0	0	1	5
No. of species	0	0	1	1
Grand total				
Abundance	0	18	218	274
No. of species	0	4	39	40

All termite mound plots (*n* = 9) were observed ten times each, a total of 2,700‐min observation for each mound treatment (i.e., 9 mounds × 10 observation periods × 30 min), while savanna plots (*n* = 9) had six replicates, producing 1,620 min of observation for both treatments (i.e., 9 savanna plots × 6 observation periods × 30 min). The complete dataset contains 520 observations of individual birds. Of these, 10 were recorded as unidentified. These 10 individuals have been removed from the table above and are not included in the data analyses.

### 3.2 Abundance, richness, and diversity on termite mounds

**Figure 3:**
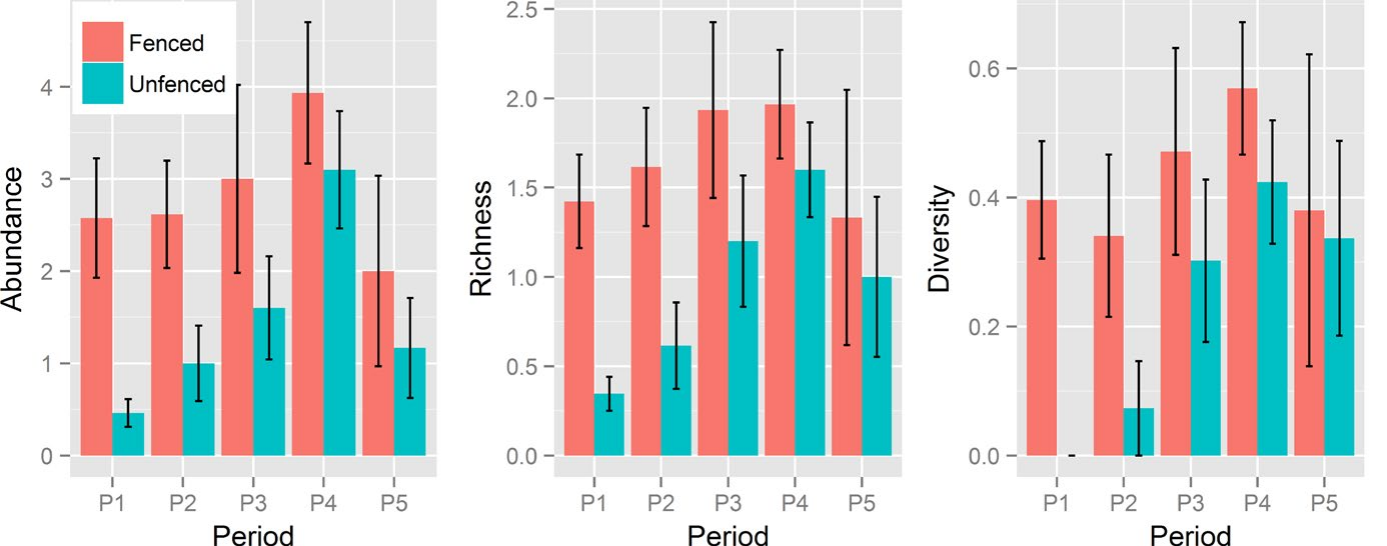
Bird abundance, richness, and diversity in five different periods throughout the season, on fenced and unfenced termite mounds. Periods [1]: 23 February–1 March, [2]: 6–9 March, [3]: 12–16 March, [4]: 20–24 March, and [5]: 17–18 April. Bars are mean values calculated from observed number of species and individuals per plot in 30‐min observation sessions. Error bars are observed standard errors. Predicted means and associated standard errors are reported in Table S2

When the influence of date and tree richness was not taken into account, the effect of fencing the savanna mounds increased abundance from 1.5 to 3.2 bird individuals and richness from 1 to 2.6 individuals per 30‐min. observation sessions (abundance [unfenced]: β = 0.40, *SE* = 0.31, [fenced]: β = 0.93, *SE* = 0.22, *z *=* *2.4, *p *=* *.015; richness [unfenced]: β = −0.027, *SE* = 0.28, [fenced]: β = 0.46, *SE* = 0.19, *z *=* *2.6, *p *=* *.0086). However, the effect of fencing depended on date (abundance [treatment × period], LRT: χ^2^ = 24.2, *df *= 1, *p *<* *.0001; richness [treatment × period] LRT: χ^2^ = 10.4, *df *= 1, *p *=* *.034, Figure 3), with the positive effect of fencing being greater in February (period 1) than in March (period 2, period 3, and period 4), the onset of the wet season. The difference in species abundance and richness between the treatments progressively decreased throughout March. The effect of fencing on the estimated difference between period 1 (late February) and period 5 (mid‐April) was similar to the difference between period 1 and period 3 (mid‐March), but the late February versus mid‐April difference was not statistically significant. Bird abundance and richness were also positively influenced by richness of tree species per plot. Although bird species diversity was also higher on fenced mounds and varied among periods (Figure 3,Table S2), the treatment × period interaction was not significant (LRT: χ^2^ = 3.2, *df* = 4, *p *=* *.52).

### 3.3 Community composition on termite mounds

**Figure 4:**
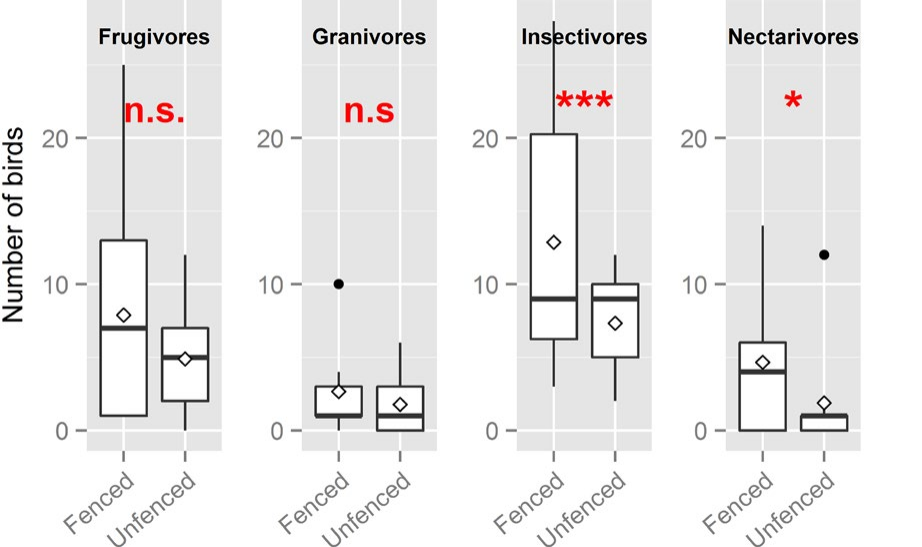
Boxplot of observed values of bird abundance in different feeding guilds. The input data are the total number of birds observed per plot, summed over ten different observation sessions (in order to increase readability)—each of 30‐min duration—within plots on fenced and unfenced termite mounds. Horizontal black lines show median, and diamonds show observed means

**Figure 5:**
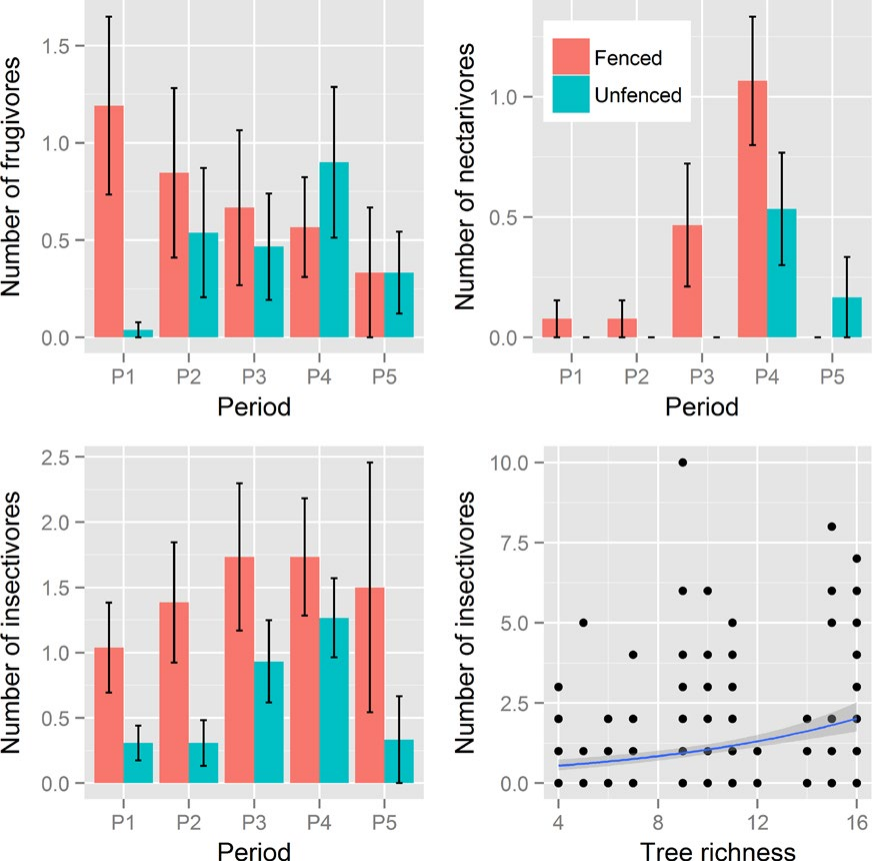
The effect of treatment and other explanatory variables (period, tree richness) on the abundance of frugivores, insectivores, and nectarivores. Bars are mean values calculated from observed number of species and individuals per plot in 30‐min observation sessions. Error bars are observed standard errors. The fitted line in the lower right panel show the estimated relationship between number of insectivores and tree richness, and the associated 95% confidence region, whereas filled circles show observed values. Predicted means and associated standard errors are reported in >Table S2

The distribution of number of bird individuals of each feeding guild category differed between fenced and unfenced termite mounds (Figure 4, Fisher's exact test: *p *=* *.02). For frugivores, fencing increased the number of individuals, but the effect depended on date (treatment × period, LRT: χ^2^ = 34.1, *df *= 4, *p *<* *.0001); the difference was largest in period 1 and decreased until period 4 (Figure 5, Table S3).

For insectivores, fencing increased the number of individuals, and the number of individuals increased from period 1 to period 4, but the effect of fencing did not depend on date (treatment × period, LRT: χ^2^ = 6.9, *df *= 4, *p *=* *.14, Figure 5, Table S3). In addition, the abundance of insectivores increased with tree richness (Figure 5, Table S3). For nectarivores, fencing increased the number of individuals, and the number of individuals was higher in period 3 and period 4 than in period 1 and period 2, but the treatment × period combination had no observations of nectarivores. We found no significant treatment differences in number of granivores (Figure 4).

Community composition was associated with tree richness (*R* ^2^ = 0.33, *p *=* *.048) but not tree density (*R* ^2^ = 0.20, *p *=* *.21) nor treatment (*R* ^2^ = 0.0063, *p *=* *.9) when fitting the environmental variables onto the NMDS axis 1 versus axis 2 ordination. No significant association between the environmental variables and the bird community was found for NMDS axis 1 versus axis 3.

### 3.4 Bird behavior on termite mounds

Overall, the relative distribution of observed bird behavior differed between fenced and unfenced termite mounds (Fisher's exact test: *p *=* *.0018, Table S4). Particularly, feeding differed between fenced and unfenced mounds (Tables S4 and S5). For feeding behavior, the representation by frugivores, granivores, insectivores, and nectarivores differed between fenced and unfenced mounds, with a higher proportion of frugivores and nectarivores feeding on fenced mounds (Fisher's Exact test: *p *<* *.00001, Table S5). For territorial behavior, there was no statistically significant difference between fenced and unfenced in the relative representation of feeding guilds (Fisher's exact test: *p *=* *.45).

## 4 DISCUSSION

Birds used the termite mounds almost exclusively compared to the savanna matrix, irrespective of guild and species. Although no birds were observed on the unfenced savanna, we did record 18 individuals, comprising only 3.5% of all the birds noted in the study, when large mammals were excluded from the savanna. Within termite mounds, mean abundance and richness of birds observed during 30‐min sessions doubled on fenced (large herbivores excluded) compared to unfenced plots. The difference was also less pronounced in the relatively wet March‐April compared with the dry February. In terms of abundance, frugivores and insectivores were the feeding guilds that profited most from herbivore exclusion.

This study has shown the substantial importance of termite mounds for bird species abundance, richness, and diversity in a savanna ecosystem. Although we predicted a greater abundance, richness, and diversity of birds on termite mounds, we did not anticipate birds to be almost exclusively associated with mounds. The mounds cover only 5% of the savanna area (Moe, Mobæk et al., 2009), but represent key resources in a grass‐dominated savanna matrix, harboring >90% of the individual birds and bird species. Dense and diverse woody vegetation (i.e., four times higher tree densities and four times as many species on mounds compared with savanna, Table 1) is mainly associated with termite mounds, whereas savanna areas have only scattered single trees. Our study shows that the complex and rich vegetation on termite mounds provides crucial resources for feeding habitat for savanna birds. Particularly insectivores birds responded strongly to mound tree richness with four times as many bird observation on mounds with high tree richness (16 tree species) compared to mounds with few trees (4 tree species). Although we are not aware of other studies that have documented the use of termite mounds by the entire local bird species assemblage, one study has shown that species richness and abundance of cavity‐using birds increase in miombo woodland areas with higher densities of *Macrotermes* mounds (Joseph et al., 2011).

Whereas termite mounds supported a rich assemblage of birds, large herbivores appeared to reduce bird abundance, richness, and diversity on termite mounds. Insectivores, nectarivores, and frugivores in particular profited from mammal exclusion. We recorded twice as many sunbirds (*Cinnyris* spp.) (nectarivores) when mammals were excluded. Of the more common species, particularly the frugivorous Rüppell's starling (*Lamprotornis purpuroptera*) and dark‐capped bulbul (*Pycnonotus tricolor*) increased in abundance (4 vs. 20 and 15 vs. 22 individuals for Rüppell's starling and dark‐capped bulbul, respectively) when large mammals were excluded from termite mounds. Our study supports findings from another experimental study, conducted in Kenya, in which excluding large herbivorous mammals increased bird diversity and abundance (Ogada et al., 2008). However, in that study, the effect of herbivory was mainly experimentally attributable to elephants and giraffes (*Giraffa camelopardalis*) (Ogada et al., 2008), two megaherbivore species that were not present in LMNP at the time of our study (although giraffes have recently been translocated to the park).

As predicted, frugivorous and nectarivorous birds increased feeding activity when large herbivores were excluded, which suggests that fenced mounds provided more or better food for these feeding guilds. We do not have any data on relationships between flower and fruit production on termite mounds and large mammal herbivory, but it is plausible that browsers clip fruit‐producing branches and shoots and consequently reduce flower and fruit production (Hendrix, 1988). Although it has also been shown that some plant species may actually increase flower and fruit production when intensively browsed (Paige & Whitham, 1985), this appears to be exceptional (see Wilkerson et al., 2013; Young & Augustine, 2007; and references therein). Intensive herbivory may also induce stress responses in plants, altering resource allocation from reproductive parts (i.e., flowers and fruits) to plant secondary metabolites (Boege & Marquis, 2005).

Of the insectivorous birds, black‐headed gonolek (*Laniarius erythrogaster*) (12 vs.19), cisticolas (*Cisticola* spp.), and other warblers (*Phylloscopus trochilus* and *Phyllolais pulchella*) (1 vs. 14) increased when large mammals were excluded. Excluding large herbivores rapidly increases canopy closure on termite mound vegetation (pers. obs.). Increased complexity of woody vegetation may promote insect production, attracting some insectivorous species that feed in the tree canopy.

Bird species richness and diversity increased from February to March. This could reflect the arrival of the rains in March, which may lead to an influx of birds starting to breed. Onset of breeding may also lead to birds becoming more conspicuous, or an increase in occupancy (amount of time each day they are on the mounds to be observed), thereby leading to more records during our observation periods. More interestingly, however, the relationship between bird species richness and treatment interacted. More species used the fenced mounds in February compared with unfenced mounds, whereas in March, the difference between fenced and unfenced mounds was significantly smaller. Thus, after the onset of the growing season, the negative effects of large mammals on bird species richness would appear to be less. By far the dominant ungulate in our study area is the mixed feeding impala, which is predominantly a browser in the dry season and a grazer in the wet season (Dunham, 1982). Mound vegetation, comprising mostly browse, is therefore likely to be more intensively used during the dry season than during the wet season.

Bird community composition was associated with tree species richness, but not tree density, nor treatment. It is not possible to account for hierarchical sampling design in ordination analysis (such as NMDS), in contrast to the GLMMs, and we think that this may be one reason why treatment did not appear to affect community composition. Interesting, however, is our findings that a species‐rich woody vegetation does seem to support different bird communities than areas with fewer tree species.

The plot sizes and distances between plots in this study were well below the home range size of any of the recorded bird species. Therefore, our data reflect how birds select microhabitats among treatments, not their restriction to individual mounds. This savanna landscape, in which termite mounds are effectively forest‐covered hot spots in a matrix of grass‐dominated savanna, is widespread, not only in Uganda (easily identified on Google Earth, Figure 1), but also in other areas in eastern and Southern Africa (e.g., Bonachela et al., 2015; Levick et al., 2010). Thus, over extensive savanna areas, *Macrotermes* termites are one of the providers of key resources for the bird communities. Had our plot sizes been larger, comprising both termite mounds and surrounding savanna, they would have masked the reality that in our study area, birds use termite mounds almost exclusively in this savanna landscape.

In conclusion, this is the first study to document a fundamental impact that *Macrotermes* termites have on bird abundance, richness, and diversity in an African savanna. Throughout African savannas, termite mounds are resource hot spots caused by termites that concentrate nutrients and increase soil turnover. These mounds are favored microsites for trees and mound vegetation that provide key resources for birds. Whereas the activities of termites substantially increase bird abundance, richness, and diversity, large herbivores reduce positive effects, although the reduction is moderate; the birdlife on unfenced termite mounds is also rich and diverse. The effect of large herbivores appears to be less in the wet season, probably because the dominant large herbivore in the area, impala, switches from browse to grass in the wet season and consequently affects woody vegetation on mounds less. Birds may play a role in the control of insect herbivory on mound vegetation, for pollination of mound vegetation, and in dispersal of seeds between mounds, but such relationships remain to be studied. However, based on the results of the present study, exclusion of large herbivores (fencing) is likely to enhance these roles both because of increased number of individuals and increased feeding activity.

The close relationship between the avifauna and termites underscores the key functional role of termites in African savannas and supports the findings of other studies on how termites influence other taxa (Fleming & Loveridge, 2003; Holdo & McDowell, 2004; Loveridge & Moe, 2004; Okullo & Moe, 2012b; Okullo et al., 2013; Pringle et al., 2010; Van der Plas et al., 2013). Birds are central to many ecosystem services (e.g., pollination, seed dispersal, and the regulation of some invertebrate populations) and are therefore crucial to savanna ecosystem structure and function. This study has shown how the role of birds in savanna dynamics depends on the distribution and abundance of termite mounds.

## CONFLICT OF INTEREST

None declared.

## AUTHOR CONTRIBUTION

SRM designed the experiment and wrote the first draft of the manuscript. KE analyzed the data and wrote the first draft of the Results chapter. OTR and OL collected the data. PO, OGS, OTR, and SD assisted in writing. All authors contributed to revisions of the manuscript.

## DATA ACCESSIBILITY

Data deposited in the Dryad repository: https://doi.org/10.5061/dryad.3f158 and upon request from stein.moe@nmbu.no.
