# Supplementary material for: Strong positive effects of termites on savanna bird abundance and diversity are amplified by large herbivore exclusion
Source: Ecol Evol. 2017 Oct 23;7(23):10079–88. doi: 10.1002/ece3.3513 (PMC5723628; doi:10.1002/ece3.3513)
Supplement: Supplementary file 1 [file ECE3-7-10079-s001.docx]

Supporting information for

**Strong positive effects of termites on savanna bird abundance and diversity are amplified by large herbivore exclusion**

Stein R. Moe^1*^, Katrine Eldegard^1^, Ole Tobias Rannestad^1^, Paul Okullo^2^, Ommund Lindtjørn^1^, Ole Gunnar Støen^1^ and Svein Dale^1^

^1^Faculty of Environmental Sciences and Natural Resource Management, Norwegian University of Life Sciences, P.O. Box 5003, NO-1432 Ås, Norway

^2^National Agricultural Research Organisation, Nabuin Zonal Agricultural Research and Development Institute, P.O. Box 132, Moroto, Uganda.

Data accessibilit y statement: Data is available on Dryad and upon request

from sanram@ucdavis.edu.

Data accessibilit y statement: Data is available on Dryad and upon request

from sanram@ucdavis.edu.

**^*^**Correspondence SRM: [stein.moe@nmbu.no](mailto:stein.moe@nmbu.no)

**Table S1.** Total number of birds observed on unfenced savanna (US), fenced savanna (FS), unfenced mound (UM) and fenced mound (FM), grouped by feeding guild and species

|  | **Treatment** | | | |
| --- | --- | --- | --- | --- |
| **Species by guilds** | **US** | **FS** | **UM** | **FM** |
| **Frugivores** |  |  |  |  |
| Bare-faced go-away-bird (*Corythaixoides personatus*) | 0 | 0 | 0 | 2 |
| Speckled mousebird (*Colius striatus*) | 0 | 0 | 13 | 14 |
| Blue-naped mousebird (*Urocolius macrourus*) | 0 | 0 | 9 | 3 |
| Spot-flanked barbet (*Tricholaema lacrymosa*) | 0 | 0 | 12 | 10 |
| Dark-capped bulbul (*Pycnonotus tricolor*) | 0 | 0 | 15 | 22 |
| Rüppell's starling (*Lamprotornis purpuroptera*) | 0 | 0 | 4 | 20 |
| **Total frugivores** | **0** | **0** | **53** | **71** |
|  |  |  |  |  |
| **Graminivores** |  |  |  |  |
| Red-eyed dove (*Streptopelia semitorquata*) | 0 | 0 | 1 | 4 |
| Ring-necked dove (*Streptopelia capicola*) | 0 | 0 | 5 | 6 |
| Spectacled weaver (*Ploceus ocularis*) | 0 | 0 | 0 | 1 |
| Holub’s golden weaver (*Ploceus xanthops*) | 0 | 0 | 2 | 2 |
| Lesser masked weaver (*Ploceus intermedius*) | 0 | 0 | 6 | 8 |
| Weaver sp. (*Ploceus* sp*.*) | 0 | 0 | 0 | 2 |
| Firefinch sp. (*Lagonosticta* sp.) | 0 | 0 | 2 | 0 |
| Yellow-fronted canary (*Crithagra mozambica*) | 0 | 0 | 3 | 1 |
| **Total graminivores** | **0** | **0** | **19** | **24** |
|  |  |  |  |  |
| **Insectivores** |  |  |  |  |
| White-browed coucal (*Centropus superciliosus*) | 0 | 0 | 0 | 1 |
| Levaillant's cuckoo (*Clamator levaillantii*) | 0 | 0 | 2 | 0 |
| Lilac-breasted roller (*Coracias caudatus*) | 0 | 0 | 2 | 2 |
| Striped kingfisher (*Halcyon chelicuti*) | 0 | 0 | 1 | 0 |
| Woodland kingfisher (*Halcyon senegalensis*) | 0 | 0 | 1 | 0 |
| Little beeater (*Merops pusillu*s) | 0 | 0 | 3 | 12 |
| Green woodhoopoe (*Phoeniculus purpureus*) | 0 | 0 | 0 | 3 |
| Bearded woodpecker (*Dendropicos namaquus*) | 0 | 0 | 3 | 1 |
| Cardinal woodpecker (*Dendropicos fuscescens*) | 0 | 0 | 0 | 1 |
| Chin-spot batis (*Batis molitor*) | 0 | 8 | 0 | 1 |
| Orange-breasted bush-shrike (*Chlorophoneus sulfureopectus*) | 0 | 0 | 1 | 2 |
| Black-headed gonolek (*Laniarius erythrogaster*) | 0 | 0 | 12 | 19 |
| Grey-backed fiscal (*Lanius excubitoroides*) | 0 | 0 | 1 | 0 |
| White-winged black-tit (*Melaniparus leucomelas*) | 0 | 0 | 3 | 0 |
| Red-faced crombec (*Sylvietta whytii*) | 0 | 0 | 1 | 0 |
| Willow warbler (*Phylloscopus trochilus*) | 0 | 0 | 6 | 4 |
| Trilling cisticola (*Cisticola woosnami*) | 0 | 3 | 5 | 7 |
| Croaking cisticola (*Cisticola natalensis*) | 0 | 0 | 8 | 0 |
| Long-tailed cisticola (*Cisticola angusticauda*) | 0 | 0 | 0 | 3 |
| Cisticola sp. (*Cisticola* sp.) | 0 | 0 | 3 | 3 |
| Tawny-flanked prinia (*Prinia subflava*) | 0 | 6 | 6 | 6 |
| Buff-bellied warbler (*Phyllolais pulchella*) | 0 | 0 | 0 | 1 |
| Yellow-breasted apalis (*Apalis flavida*) | 0 | 0 | 1 | 5 |
| Grey-backed camaroptera (*Camaroptera brevicaudata*) | 0 | 0 | 9 | 11 |
| Warbler/Cisticola sp. | 0 | 0 | 1 | 14 |
| Black-lored babbler (*Turdoides sharpei*) | 0 | 0 | 8 | 14 |
| Arrow-marked babbler (*Turdoides jardineii*) | 0 | 0 | 1 | 0 |
| White-browed robin-chat (*Cossypha heuglini*) | 0 | 0 | 36 | 14 |
| Whinchat (*Saxicola rubetra*) | 0 | 1 | 0 | 0 |
| Sooty chat (*Myrmecocichla nigra*) | 0 | 0 | 4 | 2 |
| Yellow wagtail (*Motacilla flava*) | 0 | 0 | 0 | 2 |
| Yellow-throated longclaw (*Macronyx croceus*) | 0 | 0 | 6 | 4 |
| **Total insectivores** | **0** | **18** | **124** | **132** |
|  |  |  |  |  |
| **Nectarivores** |  |  |  |  |
| Marico sunbird (*Cinnyris mariquensis*) | 0 | 0 | 14 | 33 |
| Copper sunbird (*Cinnyris cupreus*) | 0 | 0 | 3 | 8 |
| Sunbird sp. (*Cinnyris* sp.) | 0 | 0 | 4 | 1 |
| **Total nectarivores** | **0** | **0** | **21** | **42** |
|  |  |  |  |  |
| **Other** |  |  |  |  |
| Nightjar sp. (*Caprimulgus* sp.) | 0 | 0 | 1 | 0 |
| Greater honeyguide (*Indicator indicator*) | 0 | 0 | 0 | 5 |
| **Total Other** | **0** | **0** | **1** | **5** |
|  |  |  |  |  |
| **Grand total** | **0** | **18** | **218** | **274** |

NOTE: The complete dataset contains 520 observations of individual birds. Of these, 10 were recorded as Unidentified. These 10 individuals have been removed from the table above and are not included in the data analyses.

**Table S2.** Variables influencing abundance, species richness, and diversity of birds on fenced and unfenced (reference level) termite mounds. Final generalized mixed models involved model selection by backward elimination of explanatory variables. Sample unit is the number of species or individuals per plot in 30-min observation sessions. The categorical variable period has five levels: [1]: 23. February – 1. March, [2]: 6. – 9. March, [3]: 12. – 16. March, [4]: 20. – 24. March, [5]: 17. – 18. April. Explanatory variables were retained in the final models and shown in the table if their influence on a response variable was statistically significant (*P* < 0.05) or suggested a trend (*P* < 0.10) ^1^

| Explanatory variables |  |  |  |  |
| --- | --- | --- | --- | --- |
| Bird abundance (number of individuals)^2^ | |  |  |  |
| Fixed effects | Estimate | SE | Z | P |
| Intercept | -1.7 | 0.46 | -3.8 | 0.00018 |
| Treatment fenced (vs unfenced) | 1.7 | 0.36 | 4.7 | <0.0001 |
| Period 2 (vs Period 1) | 0.75 | 0.40 | 1.9 | 0.059 |
| Period 3 (vs Period 1) | 1.2 | 0.35 | 3.6 | 0.00038 |
| Period 4 (vs Period 1) | 1.9 | 0.30 | 6.2 | <0.0001 |
| Period 5 (vs Period 1) | 1.1 | 0.48 | 2.358 | 0.018 |
| Tree richness | 0.087 | 0.030 | 2.9 | 0.0033 |
| Treatment fenced × Period 2 | -0.85 | 0.45 | -1.9 | 0.061 |
| Treatment fenced × Period 3 | -1.1 | 0.40 | -2.7 | 0.0060 |
| Treatment fenced × Period 4 | -1.5 | 0.34 | -4.4 | <0.0001 |
| Treatment fenced × Period 5 | -1.1 | 0.57 | -1.9 | 0.052 |
|  |  |  |  |  |
| Plot/Site/Location | 0.13 | 0.36 |  |  |
| Site/Location | 0.033 | 0.18 |  |  |
| Location | <0.0001 | 0.0001 |  |  |
| Time of day | 0.024 | 0.15 |  |  |
| Weather | <0.0001 | 0.0024 |  |  |
| Observer | 0.024 | 0.16 |  |  |
|  |  |  |  |  |
| Bird species richness (number of species)^3^ | |  |  |  |
| Fixed effects | Estimate | SE | Z | P |
| Intercept | -1.9 | 0.42 | -4.5 | <0.0001 |
| Treatment fenced (vs unfenced) | 1.4 | 0.38 | 3.6 | 0.00035 |
| Period 2 (vs Period 1) | 0.54 | 0.49 | 1.1 | 0.27 |
| Period 3 (vs Period 1) | 1.3 | 0.41 | 3.1 | 0.0020 |
| Period 4 (vs Period 1) | 1.5 | 0.36 | 4.2 | <0.0001 |
| Period 5 (vs Period 1) | 1.2 | 0.54 | 2.3 | 0.024 |
| Tree richness | 0.080 | 0.022 | 3.7 | 0.00023 |
| Treatment fenced × Period 2 | -0.47 | 0.56 | -0.83 | 0.40 |
| Treatment fenced × Period 3 | -0.92 | 0.48 | -1.9 | 0.053 |
| Treatment fenced × Period 4 | -1.2 | 0.42 | -2.9 | 0.0038 |
| Treatment fenced × Period 5 | -1.1 | 0.66 | -1.6 | 0.10 |
| Random effects^1^ | Variance | SD |  |  |
| Plot/Site/Location | 0.032 | 0.18 |  |  |
| Site/Location | 0.013 | 0.12 |  |  |
| Location | <0.0001 | 0.00043 |  |  |
| Time of day | 0.014 | 0.12 |  |  |
| Weather | <0.0001 | 0.00037 |  |  |
| Observer | 0.0094 | 0.097 |  |  |
|  |  |  |  |  |
| Bird diversity (Shannon diversity index)^4^ | |  |  |  |
| Fixed effects | Estimate | SE | t | ^5^P |
| Intercept | -0.27 | 1.17 | -1.6 |  |
| Treatment fenced (vs unfenced) | 0.24 | 0.082 | 2.9 | 0.0055 |
| Period 2 (vs Period 1) | -0.0029 | 0.10 | -0.028 | 0.0045 |
| Period 3 (vs Period 1) | 0.17 | 0.098 | 1.7 |  |
| Period 4 (vs Period 1) | 0.28 | 0.082 | 3.4 |  |
| Period 5 (vs Period 1) | 0.27 | 0.14 | 1.9 |  |
| Tree richness | 0.043 | 0.011 | 4.0 | 0.0012 |
| Random effects^1^ | Variance | SD |  |  |
| Plot/Site/Location | 0.013 | 0.12 |  |  |
| Site/Location | 0 | 0 |  |  |
| Location | 0 | 0 |  |  |
| Time of day | 0.042 | 0.065 |  |  |
| Weather | 0.016 | 0.13 |  |  |
| Observer | 0.0035 | 0.059 |  |  |

^1^Sample size: 180 observation periods; each of the 2 treatments x 9 plots were observed 10 times. Time of day, with two levels: morning; evening. Weather, with three levels: sun; clouded; light rain. Observer, with two levels: observer 1; observer 2. ^2^Log link, negative binomial distribution of errors. ^3^Log link, Poisson distribution of errors. ^4^Identity link, normal distribution of errors. ^5^P-values from likelihood ratio tests.

**Table S3.** Variables influencing abundance of birds within different feeding guilds^1^ on fenced and unfenced (reference level) termite mounds. Final generalized mixed models involved model selection by backward elimination of explanatory variables. Sample unit is the number of species or individuals per plot in 30-min observation sessions. The categorical variable period has five levels: [1]: 23. February – 1. March, [2]: 6. – 9. March, [3]: 12. – 16. March, [4]: 20. – 24. March, [5]: 17. – 18. April. Explanatory variables were retained in the final models and shown in the table if their influence on a response variable was statistically significant (*P* < 0.05) or suggested a trend (*P* < 0.10).

| Explanatory variables |  |  |  |  |
| --- | --- | --- | --- | --- |
| Frugivore abundance (number of individuals)^1^ | |  |  |  |
| Fixed effects | Estimate | SE | Z | P |
| Intercept | -4.07 | 1.17 | -3.49 | 0.0005 |
| Treatment fenced (vs unfenced) | 3.31 | 1.03 | 3.22 | 0.0013 |
| Period 2 (vs Period 1) | 2.47 | 1.04 | 2.39 | 0.0170 |
| Period 3 (vs Period 1) | 2.68 | 1.03 | 2.60 | 0.0092 |
| Period 4 (vs Period 1) | 3.25 | 0.98 | 3.31 | 0.0009 |
| Period 5 (vs Period 1) | 2.07 | 1.20 | 1.73 | 0.0830 |
| Treatment fenced × Period 2 | -2.92 | 1.09 | -2.68 | 0.0074 |
| Treatment fenced × Period 3 | -3.18 | 1.09 | -2.92 | 0.0035 |
| Treatment fenced × Period 4 | -3.98 | 1.02 | -3.89 | 0.0001 |
| Treatment fenced × Period 5 | -3.12 | 1.38 | -2.26 | 0.0239 |
| Random effects^2^ | Variance | SD |  |  |
| Plot/Site/Location | 0.38 | 0.62 |  |  |
| Site/Location | 0.63 | 0.79 |  |  |
| Location | <0.0001 | 0.0001 |  |  |
| Weather | 0.60 | 0.76 |  |  |
| Observer | 0.022 | 0.15 |  |  |
| Time of day | 0.027 | 0.17 |  |  |
|  |  |  |  |  |
| Insectivore abundance (number of individuals)^1^ | | |  |  |
| Fixed effects | Estimate | SE | t | ^5^P |
| Intercept | -1.82 | 0.52 | -3.54 | 0.0004 |
| Treatment fenced (vs unfenced) | 0.53 | 0.23 | 2.26 | 0.024 |
| Period 2 (vs Period 1) | 0.22 | 0.28 | 0.78 | 0.43 |
| Period 3 (vs Period 1) | 0.49 | 0.25 | 2.01 | 0.044 |
| Period 4 (vs Period 1) | 0.76 | 0.21 | 3.70 | 0.00022 |
| Period 5 (vs Period 1) | 0.54 | 0.38 | 1.43 | 0.15 |
| Tree richness | 0.11 | 0.03 | 3.77 | 0.00016 |
| Random effects^2^ | Variance | SD |  |  |
| Plot/Site/Location | 0.130 | 0.361 |  |  |
| Site/Location | <0.0001 | <0.0001 |  |  |
| Location | <0.0001 | 0.001 |  |  |
| Weather | 0.073 | 0.271 |  |  |
| Observer | 0.165 | 0.406 |  |  |
| Time of day | 0.020 | 0.142 |  |  |
|  | | |  |  |
| Nectarivore abundance (number of individuals)^1,3^ | | |  |  |
| Fixed effects | Estimate | SE | Z | P |
| Intercept | -5.05 | 1.08 | -4.67 | <0.00001 |
| Treatment fenced (vs unfenced) | 1.32 | 0.73 | 1.81 | 0.071 |
| Period 2 (vs Period 1) | 0.00 | 1.23 | 0.00 | 1.0 |
| Period 3 (vs Period 1) | 1.81 | 0.80 | 2.25 | 0.024 |
| Period 4 (vs Period 1) | 3.03 | 0.73 | 4.17 | <0.00001 |
| Period 5 (vs Period 1) | 1.53 | 1.27 | 1.20 | 0.23 |
|  |  |  |  |  |
| Random effects^2^ | Variance | SD |  |  |
| Plot/Site/Location | 1.3230 | 1.1500 |  |  |
| Site/Location | 0.3141 | 0.5605 |  |  |
| Location | 0.4785 | 0.6918 |  |  |
| Weather | <0.00001 | 0.0001 |  |  |
| Observer | 0.0630 | 0.2510 |  |  |
| Time of day | 0.0674 | 0.2595 |  |  |

^1^Log link, Poisson distribution of errors. ^2^Sample size: 180 observation periods; each of the 2 treatments x 9 plots were observed 10 times. Time of day, with two levels: morning; evening. Weather, with three levels: sun; clouded; light rain. Observer, with two levels: observer 1; observer 2.

**Table S4.** Number of observations of different bird behaviours on fenced and unfenced termite mounds.

| Behaviour | Fenced | Unfenced | Total no. observations | Fenced expected* | Unfenced expected* | % Fenced |
| --- | --- | --- | --- | --- | --- | --- |
| Feeding | 73 | 39 | 112 | 66.8 | 45.2 | 65.2 |
| Locomoting | 6 | 10 | 16 | 9.5 | 6.5 | 37.5 |
| Parental** | 4 | 4 | 8 | 4.8 | 3.2 | 50.0 |
| Perching | 91 | 77 | 168 | 100.2 | 67.8 | 54.2 |
| Preening | 4 | 8 | 12 | 7.2 | 4.8 | 33.3 |
| Territorial | ***45 | ****13 | 58 | 34.6 | 23.4 | 77.6 |

*Expected values based on Chi-squared approximation; **Parental = sum of feeding offspring + nesting + visiting nest; **4 fighting + 41 singing; ***13 singing. In case of multiple observations of the same individual, we included the first entry for each individual in our field data observations.

**Table S5.** Number of observations of feeding behaviour shown by birds in the four main feeding guilds on fenced and unfenced termite mounds

| Behaviour  Guild | Fenced | Unfenced | Total no. observations | Fenced expected* | Unfenced expected* | % Fenced |
| --- | --- | --- | --- | --- | --- | --- |
| Feeding |  |  |  |  |  |  |
| Frugivores | 17 | 8 | 25 | 16.3 | 8.7 | 68.0 |
| Granivores | 4 | 5 | 9 | 5.9 | 3.1 | 44.4 |
| Insectivores | 20 | 24 | 44 | 28.7 | 15.3 | 45.5 |
| Nectarivores | 32 | 2 | 34 | 22.2 | 11.8 | 94.1 |

*Expected values based on Chi-squared approximation
